# Diagnosis Accuracy of Mean Arterial Pressure Variation during a Lung Recruitment Maneuver to Predict Fluid Responsiveness in Thoracic Surgery with One-Lung Ventilation

**DOI:** 10.1155/2016/3623710

**Published:** 2016-10-13

**Authors:** Woon-Seok Kang, Chung-Sik Oh, Chulmin Park, Bo Mi Shin, Tae-Gyoon Yoon, Ka-Young Rhee, Nam-Sik Woo, Seong-Hyop Kim

**Affiliations:** ^1^Department of Anaesthesiology and Pain Medicine, Konkuk University Medical Center, Konkuk University School of Medicine, Seoul, Republic of Korea; ^2^Research Institute of Medical Science, Konkuk University School of Medicine, Seoul, Republic of Korea

## Abstract

*Background*. Lung recruitment maneuver (LRM) during thoracic surgery can reduce systemic venous return and resulting drop in systemic blood pressure depends on the patient's fluid status. We hypothesized that changes in systemic blood pressure during the transition in LRM from one-lung ventilation (OLV) to two-lung ventilation (TLV) may provide an index to predict fluid responsiveness.* Methods*. Hemodynamic parameters were measured before LRM (*T*0); after LRM at the time of the lowest mean arterial blood pressure (MAP) (*T*1) and at 3 minutes (*T*2); before fluid administration (*T*3); and 5 minutes after ending it (*T*4). If the stroke volume index increased by >25% following 10 mL/kg colloid administration for 30 minutes, then the patients were assigned to responder group.* Results*. Changes in MAP, central venous pressure (CVP), and stroke volume variation (SVV) between *T*0 and *T*1 were significantly larger in responders. Areas under the curve for change in MAP, CVP, and SVV were 0.852, 0.759, and 0.820, respectively; the optimal threshold values for distinguishment of responders were 9.5 mmHg, 0.5 mmHg, and 3.5%, respectively.* Conclusions*. The change in the MAP associated with LRM at the OLV to TLV conversion appears to be a useful indicator of fluid responsiveness after thoracic surgery. Trial Registration. This trial is registered at Clinical Research Information Service with KCT0000774.

## 1. Introduction

According to the Frank–Starling relationship, patients on the ascending portion of the cardiac function curve should show an increase in stroke volume and cardiac output (CO) by volume expansion, whereas patients on the flat portion of the relationship are not expected to show a significant change in the stroke volume and CO with similar volume expansion [1]. Therefore, it is important to identify the exact status of the patients with respect to the Frank–Starling relationship.

Dynamic parameters such as stroke volume variation (SVV) and pulse pressure variation (PPV) have been introduced to determine the exact condition of the patients, namely, fluid responsiveness; these parameters are useful for ensuring optimal fluid therapy [2–5]. Cyclic changes in the venous return to the heart derived by positive pressure ventilation during mechanical ventilation produce SVV and PPV [6, 7]. However, accurate measurement of SVV and PPV requires certain conditions, including mechanical ventilation with an adequate tidal volume under a closed chest wall, appropriate myocardial function, no cardiac arrhythmia, valvular or shunt disease, and sufficient peripheral vascular patency [2, 3, 8].

Thoracic surgery, using thoracotomy or video-assisted thoracic surgery (VATS), is typically performed in the lateral decubitus position, which is associated with decreased pulmonary blood flow, altered lung compliance, and increased intrathoracic pressure, thereby affecting SVV and PPV accuracy [9, 10]. Moreover, the chest wall is opened during thoracic surgery.

The lung recruitment maneuver (LRM) is routinely used toward the end of thoracic surgery during the transition from one- to two-lung ventilation (OLV to TLV) to improve oxygenation and confirm the absence of air leakage [11–14]. However, expanding alveoli can compress pulmonary vessels and reduce systemic venous return to the heart, namely, preload, possibly leading to a change in various hemodynamic parameters, such as systemic blood pressure, central venous pressure (CVP), CO, stroke volume, and SVV, commensurate with the patient's fluid status [15, 16].

The aim of the present study was to identify useful parameters for fluid management in thoracic surgery patients. Herein, we hypothesized that changes in systemic blood pressure during transition in LRM from OLV to TLV may provide an index to predict fluid responsiveness.

## 2. Methods

### 2.1. Study Population

After obtaining Institutional Review Board approval (KUH1160054; May 2013) from the Institutional Review Board of the Konkuk University Medical Center (Seoul, Korea), the study was registered at http://cris.nih.go.kr (KCT0000774). Written informed consent was obtained from patients undergoing thoracic surgery under thoracotomy or VATS with OLV at a university teaching hospital between May 2013 and December 2013; patients were studied prospectively. The exclusion criteria were (1) urgent or emergency case; (2) age <18 years or >80 years; (3) an arterial partial oxygen pressure (PaO_2_)/fraction of inspired oxygen (FiO_2_) ratio < 300 mmHg before anesthesia induction; (4) impaired left and right ventricular function (ejection fraction < 40%); (5) preoperative dysrhythmia; (6) cardiac valvular or shunt disease; and (7) peripheral vascular disease. Using a parallel study design, patients were divided into responder (group R) and nonresponder (group N) groups. To determine responder or nonresponder status, colloid solution (Volulyte®, Fresenius Kabi, Bad Homberg, Germany), at 10 mL/kg of ideal body weight, was administered toward the end of the surgical procedure with patients in a supine position [17]. If stroke volume index (SVI), measured by pulse contour analysis (Vigileo-Flotrac system generation 3.02, Edwards Lifesciences, Irvine, CA, USA), increased by >25% following intravascular volume expansion, patients were assigned to the responder group [18]. All thoracic surgery was performed by the same surgeons and nurses, who were blinded to the study protocol.

### 2.2. Anesthetic Regimen

After instituting routine invasive arterial blood pressure and noninvasive patient monitoring (pulse oximetry, electrocardiography, and bispectral index), anesthesia was induced and maintained using target-controlled infusion (Orchestra Base Primea, Fresenius Vial, Brezins, France) of propofol and remifentanil; epidural anesthesia was not provided. During anesthesia maintenance, the plasma concentration of remifentanil was fixed at 10 ng/mL and the effect-site concentration of propofol was adjusted to a bispectral index value of between 40 and 60. Muscle relaxation was induced by rocuronium bolus during the monitoring of peripheral neuromuscular transmission. A central venous catheter, for perioperative monitoring and fluid administration, was inserted via the right internal jugular vein following anesthesia induction. All patients were intubated with a Univent endotracheal tube (Silbroncho, Fuji Systems Corporation, Tokyo, Japan) for OLV. The following ventilator (ADU, Datex-Ohmeda, Finland) settings were used during TLV: volume-controlled ventilation at 4 L/min, consisting of air (3 L/min) and oxygen (1 L/min); predetermined tidal volume was calculated as ideal body weight [50 (female: 45.5) + 0.91 · (Height − 152.4)] × 7 mL; respiratory rate was controlled using end-tidal carbon dioxide pressure (EtCO_2_) ranging between 35 and 40 mmHg and capnography (S/5 Compact Anesthesia Monitor, Datex-Ohmeda, Finland). No positive end-expiratory pressure (PEEP) was applied; the inspiratory/expiratory ratio = 1 : 2. Ventilator settings during OLV were identical to those used for TLV, except for a total fresh gas flow of 4 L/min, consisting of 100% oxygen. The surgical procedure was performed in the lateral position. Proper bronchial balloon position and volume for OLV were confirmed by fiberoptic bronchoscopy (LF-GP, Olympus Medical Systems, Tokyo, Japan) before and after patients were placed in the lateral position. OLV commenced immediately before surgical incision and ceased before thorax closure. Cases requiring TLV during surgery (due to a pulse oximetry < 90%) through PEEP to the dependent lung, continuous positive airway pressure (CPAP) to the nondependent lung, or peak inspiratory pressure (PIP) > 30 cmH_2_O during OLV were not included in the analysis.

During anesthesia, systemic mean blood pressure (MAP) was maintained at >60 mmHg, and the cardiac index (CI) at >2.0 L/min/m^2^, by fluid or medication (inotropes and vasopressors) administration according to hemodynamic parameters (MAP, heart rate [HR], SVV, and CI) and the discretion of the attending anesthesiologist. Crystalloid solution with plasma solution A Inj.® (CJ HealthCare, Seoul, Korea) 2 mL/kg/h was administered according to fluid maintenance requirements, redistribution, and evaporative surgical fluid losses. The attending anesthesiologist performed additional separate laboratory tests in cases of acute surgical bleeding. If hematocrit (Hct) was >30%, then colloid solution with Volulyte (Fresenius Kabi, Bad Homberg, Germany) was administered to replace blood loss and maintain stable hemodynamic status. If Hct was <30%, then transfusion was started.

### 2.3. Measurements

**Figure 1:**
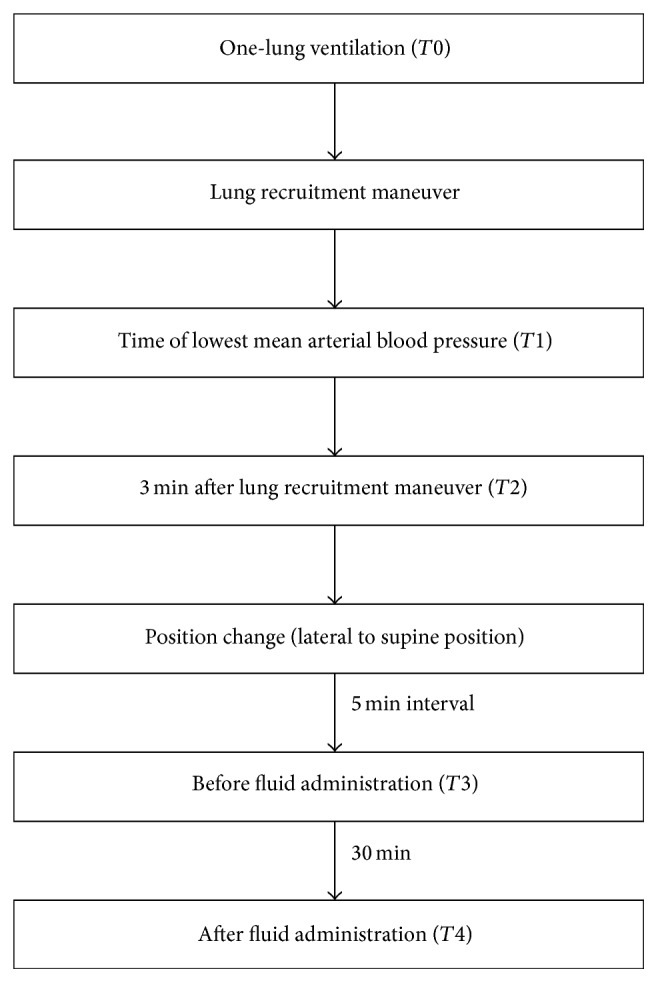
Study protocol.

Following the main surgical procedure and prior to thorax closure, OLV was ceased and LRM (the holding of one breath with TVL at 30 cmH_2_O for 10 s, repeated three times) was applied to improve oxygenation and confirm an absence of air leakage. After thorax closure, the patient was moved from the lateral to supine position, and colloid solution at 10 mL/kg of ideal body weight was administered for 30 min to confirm and determine assignment to group R or N. MAP (mmHg), HR (beats per minute), and central venous pressure (CVP, mmHg) derived by an invasive arterial and central pressure monitoring device and cardiac output (CO, L/min), CI (L/min/m^2^), SVI, and SVV (%) derived by a pulse contour analysis device were measured before (*T*0) and after LRM at the point of lowest MAP (*T*1) and at 3 min (*T*2) before administration of colloid solution at 10 mL/kg of ideal body weight (*T*3) and 5 min after its cessation (*T*4; Figure 1). During all of the measurements, there was no change in medications (e.g., inotropes and vasopressors). All of the parameters were recorded by trained observers who did not participate in the patients' care.

### 2.4. Statistics

The primary outcome variable was change in MAP from *T*0 to *T*1. A change of 7.1 ± 3.8 mmHg was calculated from the pilot study with 10 patients not included in the final analysis (confirmed as group N). For changes between *T*0 and *T*1, a minimum detected difference of 40% (MAP of 10 mmHg) among groups was considered clinically significant. A sample size of 38 in each group was calculated as appropriate to achieve a power of 0.9 and *α* value of 0.05.

Statistical analyses were conducted using the SPSS for Windows software package (ver 18.0; SPSS Inc., Chicago, IL, USA). Between-group analysis of continuous variables was performed using Student's *t*-test with the normality test or the Mann–Whitney rank-sum test. Within-group analysis of continuous variables was performed using one-way repeated measures analysis of variance of Friedman or repeated measures analysis of variance on ranks test. Categorical variables were analysed using the chi-squared test. To obtain cut-off values for changes in hemodynamic parameters (to distinguish responders and nonresponders), area under the curve (AUC), using receiver operation characteristic (ROC) curve analysis, was performed and the Youden index was used. Data are expressed as mean ± SD, median values (25%–75%), or numbers of patients. A value of *p* < 0.05 was taken to indicate statistical significance.

## 3. Results

**Figure 2:**
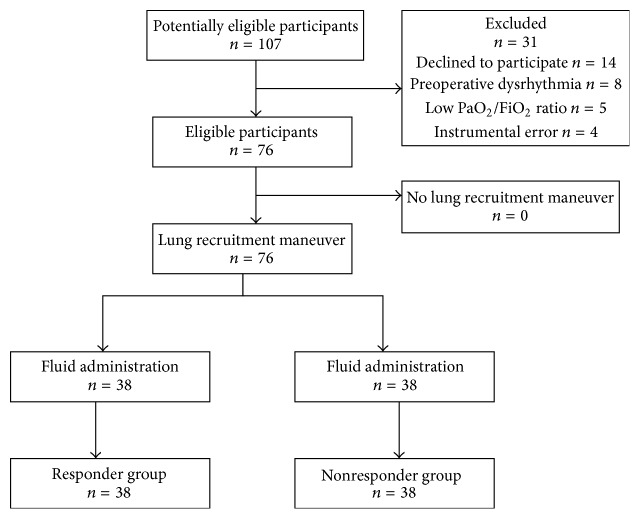
STARD flow diagram.

During the study 107 thoracic surgeries under thoracotomy or VATS with OLV were performed, with 76 patients deemed eligible for inclusion (*n* = 38 in each group) and 31 excluded; of these, 14 declined to participate, 8 exhibited preoperative dysrhythmia, and 5 were characterized by a PaO_2_/FiO_2_ ratio < 300 mmHg before anesthesia induction; in four cases there was an instrumental error (Figure 2). The study was terminated once the planned sample size was achieved; further evaluation or follow-up after discharge from the operating room was not performed. No adverse events were observed in either group.

Demographic characteristics and preoperative pulmonary function were similar among groups (Table 1), as was the amount of colloid solution administered to define responder or nonresponder status: 660 mL (range: 590–680 mL) in group R versus 680 mL (600–720 mL) in group N, *p* = 0.16.

During comparison of the hemodynamic profile of each group, MAP, HR, and CVP did not differ at any measured time point. CO and CI, at *T*0–3 in group R, were significantly lower compared with group N (CO, *p* = 0.002 at *T*0, *p* < 0.001 at *T*1, *p* = 0.01 at *T*2, *p* = 0.04 at *T*3; CI, *p* < 0.001 at *T*0, *p* < 0.001 at *T*1, *p* = 0.007 at *T*2, and *p* = 0.038 at *T*3). SVI in group R at *T*1 was significantly lower compared with group N (*p* = 0.003 at *T*1). SVV in group R at *T*1 was significantly higher compared with group N (*p* = 0.024; Table 2).

In comparisons of the hemodynamic profiles within group R, MAP, HR, CO, CI, and SVI were decreased significantly by LRM (*T*1) and increased to the before LRM level (*T*0). CVP and SVV were increased significantly at *T*1. All hemodynamic parameters at *T*1 were definitely different compared with those at *T*0. In comparisons of hemodynamic profiles within group N, MAP, CO, CI, and SVI were decreased significantly at *T*1 and *T*3. HR, CVP, and SVV were unchanged by LRM (*T*1; Table 3).

Changes in hemodynamic parameters before (*T*0) and after (*T*1) LRM, compared among groups, were calculated by subtracting values for *T*0 from *T*1. Changes in MAP, CVP, and SVV, between *T*0 and *T*1 in group R, were significantly greater compared with group N (MAP, 11.7 ± 3.4 mmHg in group R versus 6.4 ± 4.3 mmHg in group N, *p* < 0.001; CVP = 2 [1–4.5] mmHg in group R versus 0 [0-1] mmHg in group N, *p* = 0.02; SVV = 6 [2–9]% in group R versus 2 [0–5]% in group N, *p* < 0.01); however, no other value differed, especially CI and SVI (Table 4).

**Figure 3:**
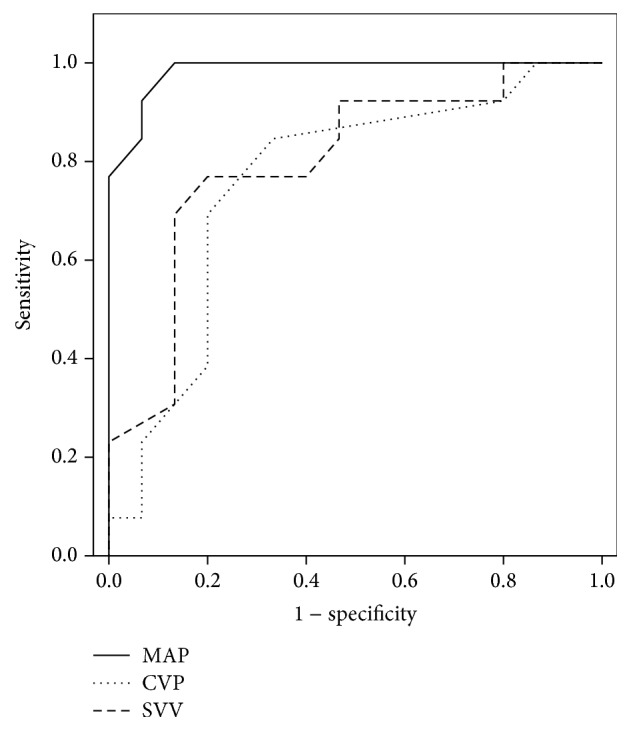
Receiver operating characteristic curve for changes in MAP, CVP, and SVV. MAP, mean arterial blood pressure; CVP, central venous pressure; SVV, stroke volume variation.

**Figure 4:**
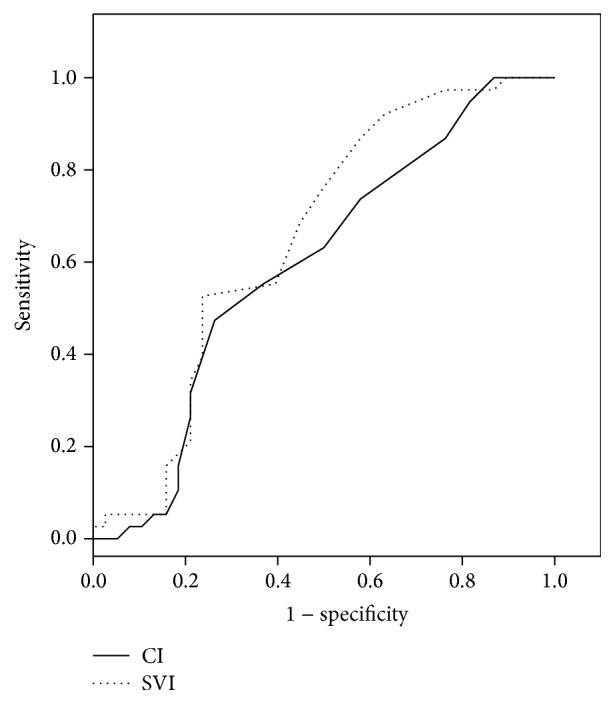
Receiver operating characteristic curve for changes in CI and SVI. CI, cardiac index; SVI, stroke volume index.

The AUCs for the change in MAP, CVP, SVV, CI, and SVI between *T*0 and *T*1 were 0.852 (95% CI: 0.767, 0.937, *p* < 0.001), 0.759 (95% CI: 0.571, 0.946, *p* = 0.02), 0.820 (95% CI: 0.724, 0.915, *p* < 0.001), 0.596 (95% CI: 0.466, 0.726, *p* = 0.15), and 0.653 (95% CI: 0.526, 0.779, *p* = 0.02), respectively. The optimal threshold values for changes in MAP, CVP, SVV, CI, and SVI between *T*0 and *T*1, used to distinguish responders from nonresponders according to the ROC curve analysis, were 9.5 mmHg [sensitivity = 73.7% (95% CI: 70.8%, 76.3%), specificity = 78.9% (95% CI: 76.2%, 81.3%)], 0.5 mmHg [sensitivity = 84.6% (95% CI: 82.2%, 86.7%), specificity = 66.7% (95% CI: 63.7%, 69.5%)], 3.5% [sensitivity 86.8% (95% CI: 84.5%, 88.7%), specificity = 65.8% (95% CI: 62.8%, 68.6%)], 0.65 L/min/m^2^ [sensitivity = 47.4% (95% CI: 44.3%, 50.4%), specificity = 73.7% (95% CI: 70.8%, 76.3%)], and 4.5 mL/m^2^/beat [sensitivity = 76.3% (95% CI: 73.5%, 78.8%), specificity = 50.0% (95% CI: 46.9%, 53.0%)], respectively (Figures 3 and 4).

## 4. Discussion

Changes in MAP, CVP, and SVV, before and after LRM during transition from OLV to TLV, differed in magnitude between responders and nonresponders (groups R and N, resp.) during thoracic surgery with OLV. However, the difference in, and cut-off value for, CVP for fluid responsiveness was insufficiently large to infer clinical applicability. Additionally, the AUC derived from ROC curve analysis for change in SVV was smaller than that observed for MAP. Therefore, changes in MAP, of approximately 10 mmHg derived during LRM, could represent a useful indicator of fluid responsiveness in patients undergoing thoracic surgery with OLV.

Fluid therapy during thoracic surgery is important for oxygenation and hemodynamic stability. First, thoracic surgery with OLV is itself a risk factor for pulmonary dysfunction due to lung manipulation and attendant risk of ischemic or reperfusion-reexpansion injury. Second, excessive intravascular volume can induce pulmonary edema and exacerbate ventilation-perfusion mismatch and insufficient oxygenation. In contrast, restrictive intraoperative fluid administration, used to prevent pulmonary dysfunction, can induce unstable hemodynamic status [19]. Generally, in thoracic anesthesia maintenance, most of anesthesiologists administrate the minimal fluid infusion to keep the patients' lung dried states, but not hypoperfusion. However, it is not easy to maintain the balance between minimal fluid infusion and adequate hemodynamic status. If there was more accurate parameter for fluid responsiveness during thoracic surgery with OLV, it might be useful for fluid management to minimize fluid administration and to avoid hypoperfusion so that the present study was conducted.

Dynamic parameters, such as SVV and the PPV-derived heart-lung interaction, have been established previously as good indicators of fluid responsiveness during thoracic surgery with OLV [18, 20]. According to one previous study, an SVV of 10.5% can discriminate responders and nonresponders during OLV [16]. However, the cut-off value for SVV of 10.5% could not be used in the present study because it had been estimated under OLV conditions and not during the transition from OLV to TLV, such that tidal volume, inspiratory pressure, and pulmonary blood flow differed, among other parameters. Therefore, change in MAP appears to be a more appropriate indicator of fluid responsiveness during the transition from OLV to TLV during LRM, although dynamic parameters such as SVV and PPV are more suitable for the prediction of fluid responsiveness compared with static parameters. In the present study, at *T*0, SVV was <10.5% for both groups, which is notable because both were considered nonresponders during OLV. This latter observation indicates that volume status following transition from OLV to TLV, during thoracic surgery with OLV, might be inadequate, although adequate volume status was maintained with SVV during OLV. For both groups at *T*2, in the lateral position with TLV, and at *T*3 in the supine position with TLV, the increase in SVV was limited compared with *T*0 in the lateral position with OLV. Therefore, additional fluid management is not necessary according to the values of SVV because the increase in SVV was limited and within the normal range.

There are some reasons to use a low SVV cut-off value to discriminate between responders and nonresponders during OLV versus TLV [17, 21, 22]. First, a shunt flow of 20–30% typically occurs through the nonventilated lung only during OLV [9]. Intravascular volume in the nonventilated lung cannot contribute to the generation of respiratory cyclic variation of stroke volume and pulse pressure because there is no ventilation in the shunt. Therefore, shunt flow can decrease absolute values of SVV and PPV. Second, the ventilator setting for tidal volume during OLV is usually decreased to prevent barotraumas [12, 13]. The effects of tidal volume on fluid responsiveness parameters have been evaluated previously [3, 23–25]. Although the degree of accuracy remains controversial, it is clear that absolute values decreased when tidal volume was <8 mL/kg. Therefore, the low SVV values in both groups at *T*0 observed herein might be associated with the low tidal volume of 7 mL/kg.

Several other caveats are discussed presently, the first of which pertains to patients' position. Generally, large blood vessels (i.e., the superior and inferior vena cava) involved in venous return to the heart are located on the right side of the thorax; the influence of lung inflation by LRM on LVSV might differ according to whether the operation site is on the right or left. However, in our patients the operation sites were distributed similarly in both groups, which might have limited any effect of operation site. Second, there was no data about fluid balance during surgery before LRM. However, the hemodynamic profiles in both groups were in normal range and stable before LRM (*T*0). The anesthesia during surgery was maintained by the same attending anesthesiologist with constant guideline (MAP > 60 mmHg, CI > 2.0 L/min/m^2^) so that the intraoperative fluid management might be similar in both groups. Finally, LRM was applied to patients toward the end of the operation, when OLV was converted to TLV, such that the technique did not facilitate fluid management during the major part of the operation. However, LRM was a good predictor of fluid responsiveness; therefore, if employed during operations, this technique could aid fluid management. The standardization of LRM was crucial. Because it was performed on the patients with good lung compliance, not poor lung compliance, by a single anesthesiologist, the effect on the heart was not different.

We evaluated the cardiac performance and SVV using arterial pulse contour analysis. Although the gold-standard method of measuring cardiac performance is the thermodilution technique, numerous evidences have supported that it can be substituted by arterial pulse contour analysis [26, 27]. It was remarkable that there were no significant differences in the changes of CI and SVI before and after LRM between two groups, showing the significant differences in the changes of MAP, CVP, and SVV.

As mentioned previously, it had been established that excessive perioperative fluid administration was risk factor of postoperative respiratory dysfunction and mortality in thoracic surgery so that the restrictive fluid management has been considered as standard therapy [28–31]. However, it was not clear whether degree of restrictive fluid management is appropriate to avoid pulmonary edema and to maintain stable hemodynamic status. In the present study, the clinical impact was that LRM would be helpful to find out the responder group and adequate parameter for optimal fluid management. Therefore, intermittently applying the LRM during thoracic surgery with OLV under restrictive fluid management would be useful tool for intraoperative volume strategy.

In conclusion, changes in MAP before and after LRM, during transition from OLV to TLV, might be a useful predictor of fluid responsiveness in thoracic surgery with OLV; the optimal threshold value to discriminate between responders and nonresponders was 9.5 mmHg.

## Figures and Tables

**Table 1:** Comparison of the demographic characteristics and pulmonary function of responders and nonresponders.

	Group R	Group N	*p* value
Gender (male/female)	31/7	30/8	0.773
Age (y)	46 ± 19	41 ± 18	0.225
Height (cm)	167.8 ± 8.0	170.2 ± 8.9	0.212
Weight (kg)	62.4 ± 9.6	61.9 ± 9.5	0.822
Operation			
Wedge/lobectomy	23/15	24/14	0.813
Thoracotomy/VATS	9/29	8/30	0.783
Site (right/left)	18/20	16/22	0.645
PFT			
FVC	3.8 ± 0.8	3.3 ± 0.6	0.127
FEV1	2.7 ± 0.5	2.5 ± 0.5	0.366
FEV1/FVC ratio	72.2 ± 10.0	75.4 ± 9.6	0.371
FEF25–75	2.0 ± 0.9	2.2 ± 1.0	0.546

Values are expressed as numbers of patients, means ± SD, or median values (25%–75%).

Group R, responder group; group N, nonresponder group; M, male; F, female; Wedge, wedge resection; VATS, video-assisted thoracic surgery; PFT, pulmonary function test; FVC, forced vital capacity; FEV1, forced expiratory volume during 1 s; FEF25–75, forced expiratory flow (25–75%).

**Table 2:** Comparison of the hemodynamic profiles of responders and nonresponders.

	Group R					Group N				
	*T*0	*T*1	*T*2	*T*3	*T*4	*T*0	*T*1	*T*2	*T*3	*T*4
MAP	77.0 (72.0–84.0)	65.5 (60.0–70.0)	75.0 (62.0–81.0)	70.9 ± 10.7	76.7 ± 9.5	74.0 (69.0–80.0)	67.0 (62.0–73.0)	70.5 (68.0–78.0)	69.9 ± 8.3	72.7 ± 8.7
HR	61.5 (57.0–70.0)	59.0 (55.0–66.0)	61.0 (57.0–68.0)	59.0 (57.0–65.0)	57.0 (54.0–63.0)	65.5 (58.0–80.0)	64.0 (58.0–76.0)	65.5 (58.0–78.0)	62.5 (56.0–73.0)	57.0 (53.0–67.0)
CVP	6.5 (6.0–8.0)	9.0 (7.0–11.0)	7.5 (6.0–9.0)	5.5 (4.0–7.0)	7.2 ± 2.9	6.0 (2.3–8.3)	6.0 (5.0–9.8)	6.0 (2.3–8.5)	6.0 (3.3–9.0)	7.7 ± 3.8
CO	4.4 (3.9–5.2)^*∗*^	3.4 (2.3–4.2)^*∗*^	4.3 (3.6–5.2)^*∗*^	3.9 (3.3–4.9)^*∗*^	4.9 (4.3–6.3)	5.6 (4.6–6.2)	4.7 (3.5–5.2)	5.2 (4.0–6.2)	4.7 (3.7–5.3)	4.5 (3.9–5.3)
CI	2.5 (2.2–3.4)^*∗*^	2.0 (1.3–2.5)^*∗*^	2.5 (2.1–3.4)^*∗*^	2.4 (2.0–3.0)^*∗*^	2.9 (2.6–3.5)	3.3 (2.7–4.1)	2.8 (2.2–3.2)	3.2 (2.6–3.8)	2.8 (2.3–3.3)	2.6 (2.2–3.2)
SVI	41.5 (34.0–50.0)	33.0 (24.0–42.0)^*∗*^	41.5 (32.0–48.0)	37.0 (29.0–44.0)	46.5 (40.0–57.0)	46.5 (39.0–55.0)	40.5 (36.0–46.0)	43.5 (39.0–56.0)	40.5 (37.0–53.0)	43.0 (37.0–51.0)
SVV	7.0 (6.0–9.0)	12.5 (8.0–16.0)^*∗*^	8.0 (6.0–10.0)	9.0 (7.0–12.0)	7.0 (5.0–8.0)	7.5 (5.0–10.0)	9.5 (7.0–11.0)	8.0 (6.0–11.0)	9.0 (8.0–11.0)	8.0 (6.0–9.0)

Values are expressed as median values (25%–75%) or means ± SD.

^*∗*^ *p* < 0.05 compared with group N.

Group R, responder group; group N, nonresponder group; *T*0, before the lung recruitment maneuver (LRM); *T*1, at the point of lowest MAP after LRM; *T*2, 3 min after LRM; *T*3, prior to administration of colloid solution at 10 mL/kg^−1^ of ideal body weight; *T*4, 5 min following cessation of colloid solution administration; MAP, mean arterial blood pressure; HR, heart rate; CVP, central venous pressure; CO, cardiac output; CI, cardiac index; SVI, stroke volume index; SVV, stroke volume variation.

**Table 3:** Comparison of the hemodynamic profiles within groups.

	Group R					Group N				
	*T*0	*T*1	*T*2	*T*3	*T*4	*T*0	*T*1	*T*2	*T*3	*T*4
MAP	77.0 (72.0–84.0)	65.5 (60.0–70.0)^*∗*^	75.0 (62.0–81.0)^*∗*†^	72.5 (63.0–76.0)^*∗*^	78.5 (71.0–82.0)^†§^	76.1 ± 10.6	69.7 ± 10.7^*∗*^	73.1 ± 9.9	69.9 ± 8.3^*∗*^	72.7 ± 8.7
HR	61.5 (57.0–70.0)	59.0 (55.0–66.0)^*∗*^	61.0 (57.0–68.0)	59.0 (57.0–65.0)^*∗*^	57.0 (54.0–63.0)^*∗*‡^	65.5 (58.0–80.0)	64.0 (58.0–76.0)	65.5 (58.0–78.0)	62.5 (56.0–73.0)^*∗*‡^	57.0 (53.0–67.0)^*∗*†‡§^
CVP	6.9 ± 2.4	9.8 ± 4.5^*∗*^	7.5 ± 2.6	5.5 ± 2.7^†^	7.2 ± 2.9^†^	6.0 ± 4.2	6.7 ± 3.8	5.9 ± 4.3	6.3 ± 3.8	7.7 ± 3.8
CO	4.4 (3.9–5.2)	3.4 (2.3–4.2)^*∗*^	4.3 (3.6–5.2)^†^	3.9 (3.3–4.9)^†^	4.9 (4.3–6.3)^†‡§^	5.6 (4.6–6.2)	4.7 (3.5–5.2)^*∗*^	5.2 (4.0–6.2)^†^	4.7 (3.7–5.3)^*∗*^	4.5 (3.9–5.3)^*∗*‡^
CI	2.5 (2.2–3.4)	2.0 (1.3–2.5)^*∗*^	2.5 (2.1–3.4)^†^	2.4 (2.0–3.0)^†^	2.9 (2.6–3.5)^†‡§^	3.3 (2.7–4.1)	2.8 (2.2–3.2)^*∗*^	3.2 (2.6–3.8)^†^	2.8 (2.3–3.3)^*∗*^	2.6 (2.2–3.2)^*∗*‡^
SVI	41.5 (34.0–50.0)	33.0 (24.0–42.0)^*∗*^	41.5 (32.0–48.0)^†^	37.0 (29.0–44.0)^*∗*†^	46.5 (40.0–57.0)^†‡§^	46.5 (39.0–55.0)	40.5 (36.0–46.0)^*∗*^	43.5 (39.0–56.0)^†^	40.5 (37.0–53.0)^*∗*^	43.0 (37.0–51.0)^†^
SVV	7.0 (6.0–9.0)	12.5 (8.0–16.0)^*∗*^	8.0 (6.0–10.0)^†^	9.0 (7.0–12.0)^*∗*^	7.0 (5.0–8.0)^†§^	7.5 (5.0–10.0)	9.5 (7.0–11.0)	8.0 (6.0–11.0)	9.0 (8.0–11.0)^*∗*^	8.0 (6.0–9.0)

Values are expressed as median values (25%–75%) or means ± SD.

^*∗*^ *p* < 0.05 compared with *T*0, ^†^ *p* < 0.05 compared with *T*1, ^‡^ *p* < 0.05 compared with *T*2, and ^§^ *p* < 0.05 compared with *T*3.

Group R, responder group; group N, nonresponder group; *T*0, before the lung recruitment maneuver (LRM); *T*1, at the point of lowest MAP after LRM; *T*2, 3 min after LRM; *T*3, prior to administration of colloid solution at 10 mL/kg^−1^ of ideal body weight; *T*4, 5 min following cessation of colloid solution administration; MAP, mean arterial blood pressure; HR, heart rate; CVP, central venous pressure; CO, cardiac output; CI, cardiac index; SVI, stroke volume index; SVV, stroke volume variation.

**Table 4:** Comparison of changes in haemodynamic parameters before (*T*0) and after (*T*1) the lung recruitment maneuver in responders and nonresponders.

	Group R	Group N	*p* value
MAP	11.7 ± 3.4^*∗*^	6.4 ± 4.3	<0.01
HR	2.7 ± 5.0	3.2 ± 5.1	0.70
CVP	2.0 (1.0–4.5)^*∗*^	0.0 (0.0–1.0)	0.02
CO	1.1 (0.6–1.5)	0.7 (0.4–1.0)	0.07
CI	0.6 (0.3–1.0)	0.5 (0.3–0.7)	0.16
SVV	6.0 (2.0–9.0)^*∗*^	2.0 (0.0–5.0)	<0.01
SVI	9.3 ± 6.9	6.7 ± 8.0	0.133

Values are expressed as means ± SD or median values (25%–75%).

^*∗*^ *p* < 0.05 compared with group N.

Group R, responder group; group N, nonresponder group; MAP, mean arterial blood pressure; HR, heart rate; CVP, central venous pressure; CO, cardiac output; CI, cardiac index; SVV, stroke volume variation; SVI, stroke volume index.

## Abbreviations

AUC: Area under curve
CI: Cardiac index
CO: Cardiac output
CPAP: Continuous positive airway pressure
CVP: Central venous pressure
EtCO_2_: Carbon dioxide pressure
FiO_2_: Fraction of inspired oxygen
Group N: Nonresponder group
Group R: Responder group
HR: Heart rate
LRM: Lung recruitment maneuver
LVSV: Left ventricular stroke volume
MAP: Systemic mean blood pressure
OLV: One-lung ventilation
PaO_2_: Arterial partial pressure of oxygen
PEEP: Positive end-expiratory pressure
PIP: Peak inspiratory pressure
PPV: Pulse pressure variation
ROC: Receiver operation characteristic
SVI: Stroke volume index
SVV: Stroke volume variation
TLV: Two-lung ventilation
VATS: Video-assisted thoracic surgery.
